# Chromosome instability in fibroblasts derived from Li–Fraumeni syndrome families without *TP53* mutations

**DOI:** 10.1054/bjoc.2000.1444

**Published:** 2000-11

**Authors:** J M Boyle, A Spreadborough, M J Greaves, J M Birch, D Scott

**Affiliations:** CRC Cancer Genetics Group, Paterson Institute for Cancer Research, CRC Christie Research Centre, Manchester, M20 4BX; CRC Paediatric and Familial Cancer Research Group, Royal Manchester Children’s Hospital, Manchester, M27 1HA, UK

**Keywords:** Li-Fraumeni, p53, longevity, chromosome instability

## Abstract

The mean in vitro lifespan of dermal fibroblast strains derived from cancer-affected individuals belonging to families conforming to the classical Li-Fraumeni-syndrome or the Li-Fraumeni-like syndrome (LF strains), but in whom no *TP53* mutation has been found, was not significantly different to that of normal strains. This was in contrast to LF strains that carry *TP53* mutations. Cytogenetic observations of numerical and structural chromosome abnormalities were made on Giemsa stained metaphases prepared at different times during the lifespan of strains. Five strains from different LF families showed significantly increased frequencies of abnormal cells during the last 10% of their lifetime compared with seven normal strains and three other LF strains fell outside the normal range but did not reach significance. Two LF strains fell within the normal range indicating heterogeneity of the phenotype in this subset of LF fibroblasts. Numerical aberrations were the major aberration type observed. These observations of genetic instability are similar, but generally less strongly expressed, to those seen in LF strains with *TP53* mutations. The basis for genetic instability in LF strains without *TP53* mutations is not known, but appears not to involve defects in either the G _1_ checkpoint or the checkpoint kinase *hChk2*. © 2000 Cancer Research Campaign


*British Journal of Cancer* (2000) **83**(9), 1136–1138
© 2000 Cancer Research Campaign
doi: 10.1054/ bjoc.2000.1444, available online at http://www.idealibrary.com on IDEAL®

Short Communication

### Chromosome instability in fibroblasts derived from Li–Fraumeni syndrome families without *TP53* mutations

**JM Boyle[1], A Spreadborough[1], MJ Greaves[1], JM Birch[2] and D Scott[1]**

[1]CRC Cancer Genetics Group, Paterson Institute for Cancer Research, CRC Christie Research Centre, Manchester M20 4BX; [2]CRC Paediatric and Familial Cancer Research Group, Royal Manchester Children's Hospital, Manchester M27 1HA, UK

**Summary** The mean in vitro lifespan of dermal fibroblast strains derived from cancer-affected individuals belonging to families conforming to the classical Li-Fraumeni-syndrome or the Li-Fraumeni-like syndrome (LF strains), but in whom no *TP53* mutation has been found, was not significantly different to that of normal strains. This was in contrast to LF strains that carry *TP53* mutations. Cytogenetic observations of numerical and structural chromosome abnormalities were made on Giemsa stained metaphases prepared at different times during the lifespan of strains. Five strains from different LF families showed significantly increased frequencies of abnormal cells during the last 10% of their lifetime compared with seven normal strains and three other LF strains fell outside the normal range but did not reach significance. Two LF strains fell within the normal range indicating heterogeneity of the phenotype in this subset of LF fibroblasts. Numerical aberrations were the major aberration type observed. These observations of genetic instability are similar, but generally less strongly expressed, to those seen in LF strains with *TP53* mutations. The basis for genetic instability in LF strains without *TP53* mutations is not known, but appears not to involve defects in either the $G_1$ checkpoint or the checkpoint kinase *hChk2.* © 2000 Cancer Research Campaign

**Keywords:** Li-Fraumeni; p53; longevity; chromosome instability

Chromosome instability is a well documented feature of late passage cultured fibroblasts derived from families conforming to the classical Li–Fraumeni syndrome (LFS) or the Li–Fraumeni-like syndrome (LFL) and carrying heterozygous (mut/wt) germline mutations in *TP53*, the tumour suppressor gene encoding the transcription factor p53 (Bischoff et al, 1990). Indeed, we found chromosome instability to be a stronger characteristic of these strains than other features such as increased longevity and resistance to ionizing radiation damage (Boyle et al, 1998). Chromosome instability was expressed as numerical and structural chromosome changes and was temporally associated with loss of the wtp53 allele in these strains. In three strains derived from two Li–Fraumeni families in which no *TP53* mutation has been found, we also observed chromosome instability accumulating as cultures approached the end of their lifespan.

We have now enlarged this study to 11 strains from 10 different LFS or LFL families (collectively designated LF families) in which no *TP53* mutation was found despite direct sequencing of all exons, intron–exon boundaries, 3′ and 5′ untranslated regions and the promoter region (Varley et al, 1997) (Table 1). With the exception of 121MA and 142MA, all strains were derived from patients with experience of cancers typical of those in LF families (e.g. sarcomas and breast cancer). Strain 121MA was included from a cancer-unaffected brother of the cancer-affected donor of 122MA (family 119) because cells from the latter strain senesced very rapidly and it was not possible to obtain metaphases in the last 10% of its lifespan. Strain 142MA was the only fibroblast strain available from family 96. It was derived from a cancer-unaffected male, whose sister and mother had typical LFS tumours (osteosarcoma and leiomyosarcoma respectively), and although incompletely sequenced (footnote, Table 1) had behaved as a non-*TP53* mutation carrier in several functional assays.

Strains were grown from the earliest available stocks in Minimal Essential Medium plus 15% foetal calf serum (Gibco) and repeatedly split 1:3 at confluence until senescent. The maximum number of apparent population doublings was calculated for each strain. Metaphase preparations were made at intervals according to standard methods and were stained with Giemsa. All metaphases were scored for numerical aberrations and those with a near diploid chromosome number (45 or 46 chromosomes) were karyotyped and also scored for unstable structural chromosome aberrations. Accurate chromosome counts were made up to 80 chromosomes; cells with > 80 were classified as 'polyploid'.

At each sampling time the percentage of the maximum number of population doublings (PD max in Table 1) was calculated for each strain (PD% in Table 2). The normal range of each parameter was defined by seven strains from normal healthy donors sampled between 88–100% of their maximum PD. These were compared with LF strains sampled in the last 10% of their lifespan. Aberration yields for individual LF strains were considered to be significantly above normals if they exceeded the normal mean plus three standard deviations.

The mean ± SD lifespan of the nine LF strains from cancer-affected individuals (Table 1) was 30.7 ± 12.9 PD compared to either 30.3 ± 7.1 for the seven normal strains in this study (Mann–Whitney U test, $P = 0.96$) or 26.6 ± 17.0 PD for a larger group of 18 normals ($P = 0.29$). This confirms our earlier observation that the in vitro lifespans of LF strains without *TP53* mutations are not significantly different to those of normal strains, in contrast to *TP53* mutation strains (Boyle et al 1998).

An abnormal cell was defined as one not having a normal set of 46 chromosomes, i.e. one showing either numerical or structural changes or both. Because only solid staining (Giemsa) was used,

*Received 28 February 2000*
*Revised 29 June 2000*
*Accepted 11 July 2000*

*Correspondence to:* JM Boyle

**Table 1** Origin and clinical characteristics of the fibroblast strains

| Strain | Family[a] | Person | Type | Cancer experience | Sex | Age at biopsy | Therapy[b] | PD[c] max |
|---|---|---|---|---|---|---|---|---|
| 79MA | 81 | III-5 | LFS | Affected | F | 70 | No | 38 |
| 107MA | 353 | III-3 | LFL | Affected | F | 35 | No | 31 |
| 115MA | 82 | IV-5 | LFS | Affected | M | 22 | Yes (7 y) | 18 |
| 121MA | 119 | III-2 | LFS | Unaffected | M | 43 | No | 24 |
| 122MA | 119 | III-1 | LFS | Affected | M | 46 | Yes (13 y) | 15 |
| 126MA | 88 | II-2 | LFS | Affected | M | 29 | No | 53 |
| 127MA | 338 | IV-3 | LFL | Affected | F | 39 | Yes (4 mo) | 36 |
| 128MA | 348 | III-2 | LFL | Affected | F | 43 | Yes (14 y) | 43 |
| 140MA | 21 | | LFS | Affected | F | 14 | Yes (7 y) | 23 |
| 142MA | 96[d] | | LFS | Unaffected | M | 32 | No | 23 |
| 159MA | 80 | | LFS | Affected | F | 46 | Yes (15 y) | 19 |

[a]Details of family, person, type and cancer experience are given in Varley et al (1997); [b]whether or not radio- or chemo- therapy was received (number of years) before biopsy; [c]Maximum number of population doublings achieved in vitro prior to cell senescence; [d]Complete analysis of *TP53* sequence in this family has not been possible due to the poor quality of DNA obtained from biopsy material, the mutation status is therefore unknown

**Table 2** Chromosome aberrations in normal and *TP53* non-mutation Li-Fraumeni fibroblasts

| Strain | Family | PD max | PD (%) | Cells scored | % Aneuploid | | | % Polyploid | Unstable aberrations per 100 cells[a] | | | | | | | Abnormal cells (%) |
|---|---|---|---|---|---|---|---|---|---|---|---|---|---|---|---|---|
| | | | | | Total | Hypo | Hyper | | Dics | Ace | Ring | Cte | Ctm | Ctb/g | Total | |
| **Normals[b]** | **Mean ± standard deviation** | | | | **18.6 ± 3.9** | **18.3 ± 3.9** | **1.7 ± 3.7** | **1.3 ± 2.2** | **0.6 ± 1.0** | **1.7 ± 2.1** | **1.0 ± 1.3** | **0** | **0** | **5.1 ± 2.8** | **8.4 ± 2.4** | **22.0 ± 8.0** |
| **(*n* = 7)** | | | **PD% = 88–100** | | | | | | | | | | | | | |
| | | | | **Range** | **14–24** | **14–24** | **0–10** | **0–6** | **0–2** | **0–4** | **0–3** | **0** | **0** | **0–8** | **4–12** | **10.3–31.6** |
| 79MA | 81 | 38 | 30 | 54 | 18.5 | 18.5 | 0 | 0 | 0 | 0 | 0 | 0 | **2** | 6 | 8 | 24.1 |
| | | | 87 | 52 | 12 | 10 | 2 | 0 | 2 | 2 | 0 | 0 | **2** | 4 | 10 | 21.2 |
| | | | 98 | 55 | 18 | 16 | 2 | 0 | 0 | 0 | 0 | 0 | **4** | 4 | 8 | 27.3 |
| 107MA | 353 | 31 | 42 | 57 | 19 | 19 | 0 | 0 | 0 | 0 | 0 | 0 | **2** | 0 | 6 | 24.6 |
| | | | 59 | 57 | 21 | 21 | 0 | 0 | 0 | 0 | 0 | 0 | 0 | 2 | 2 | 22.8 |
| | | | 98 | 69 | 30 | 28 | 3 | 3 | 0 | 0 | 0 | 0 | 0 | 8 | 8 | 36.2 |
| 115MA | 82 | 18 | 50 | 63 | **36** | **30** | 6 | 4 | 0 | 2 | 0 | 0 | 0 | 4 | 6 | 91.7[c] |
| | | | 52 | 60 | 27 | 27 | 0 | 0 | 0 | 0 | 0 | 0 | 0 | 8 | 8 | 91.7[c] |
| | | | 77 | 54 | 15 | 11 | 4 | 0 | 0 | 0 | 0 | 0 | **2** | 8 | 10 | 100.0[d] |
| | | | 86 | 77 | **47** | **42** | 6 | 4 | 0 | 4 | 0 | 0 | 0 | **2** | 8 | 100.0[d] |
| | | | 100 | 62 | **32** | 29 | 3 | 0 | 0 | 2 | 0 | 0 | 0 | 6 | 8 | 94.0[c] |
| 121MA | 119 | 24 | 37 | 59 | 27 | 25 | 2 | 2 | 0 | 2 | 0 | 0 | 0 | 4 | 6 | 33.0 |
| | | | 94 | 64 | **36** | 27 | 9 | 3 | 0 | 4 | 0 | 0 | **2** | 2 | 8 | 43.9 |
| 122MA | 119 | 15 | 67 | 56 | 24 | 21 | 3 | 3 | 0 | 2 | 0 | 0 | 0 | 2 | 4 | 27.6 |
| 126MA[b] | 88 | 53 | 28 | 57 | 15 | 13 | 2 | 2 | 0 | 0 | 0 | 0 | 0 | 10 | 10 | 31.0 |
| | | | 76 | 47 | 15 | 13 | 2 | 0 | 0 | 0 | 0 | 0 | 0 | 4 | 4 | 19.1 |
| | | | 99 | 10 | **90** | **30** | **60** | 0 | **280** | **50** | **40** | **10** | **60** | **40** | **480** | **100.0** |
| 127MA | 338 | 36 | 23 | 54 | 11 | 11 | 0 | 0 | 0 | 4 | 0 | 0 | 0 | 2 | 6 | 16.7 |
| | | | 56 | 57 | 25 | 25 | 0 | 2 | 0 | **8** | 0 | **2** | 0 | 6 | **16** | 35.1 |
| | | | 83 | 70 | **41** | **41** | 0 | **11** | 0 | **10** | 0 | 0 | **2** | 10 | **22** | **52.9** |
| | | | 100 | 50 | 74 | 74 | 0 | 22 | 11.1 | 11.1 | 0 | 0 | 0 | 5.6 | 27.8 | 89.0 |
| 128MA | 348 | 43 | 23 | 60 | 26 | 25 | 1 | 1 | 0 | 2 | 0 | 0 | 0 | 6 | 8 | 32.6 |
| | | | 98 | 61 | **34** | **30** | 5 | 5 | 0 | 0 | 0 | 0 | 0 | 8 | 8 | 39.0 |
| 140MA | 21 | 23 | 39 | 53 | 13 | 13 | 0 | 0 | 0 | 0 | 0 | 0 | 0 | 4 | 4 | **92.0[c]** |
| | | | 91 | 64 | **99** | **97** | 2 | 0 | 0 | 8 | 0 | 0 | **4** | 4 | **16** | **100.0[c]** |
| 142MA | 96 | 23 | 52 | 55 | 24 | 24 | 0 | 0 | 0 | 0 | 0 | 0 | 0 | **14** | 14 | **93.0[f]** |
| | | | 97 | 98 | **95** | **82** | **13** | **10** | **4** | **20** | 2 | 0 | **2** | 2 | **30** | **95.0** |
| 159MA[b] | 80 | 19 | 100 | 50 | 26 | 16 | 10 | 0 | **12** | 0 | 0 | 0 | 0 | 2 | 14 | 26.0 |

[a]Scored in cells with 45 or 46 chromosomes; standard abbreviations according to ISCN (1995) Disc = dicentrics; Ace = acentrics; Ring = ring chromosomes; Cte = chromatid exchange; Ctm = minute chromosome; Ctb/g = chromatid breaks and gaps; [b]Data from Boyle et al (1998) and JMB (unpublished); [c]Majority of cells had 45 or 46 chromosomes, but most had abnormal karyotypes; [d]Majority of cells had 45, 46 or 47 chromosomes, but most had abnormal karyotypes; [e]Only one cell with 46 chromosomes (abnormal), 49 cells with 45 chromosomes; [f]Only 4/50 cells scored as ?normal, remainder with losses and gains of whole chromosomes; Values in bold are greater than normal mean + 3 SD

only the numerical and *unstable* aberrations (readily detected after solid staining) are tabulated in Table 2. However, in the 'Abnormal cells' column we have included additional, easily identifiable, stable changes such as marker chromosomes, trisomies and monosomies. Further details of these are given as footnotes. The frequencies of abnormal cells in the last 10% of the lifespan of five of 10 LF strains (115MA, 126MA, 127MA, 140MA, 142MA) were significantly greater than normals, and values of three others (107MA 121MA, 128MA) fell outside the range of normal controls but did not reach significance. Exceptions that fell within

© 2000 Cancer Research Campaign the normal range were 79MA and 159MA at > 90% PD. Although the frequency of abnormal cells in strain 122MA at 67% PD (the latest successful sampling time, see above) was within the normal range, that of strain 121MA, from an unaffected blood relative, was outside the normal range at early (37% PD) and late (94% PD) population doublings.

Numerical changes were the major aberration type observed. The relatively high frequency of hypoploidy in normals (mean 18.6%) may have included cells that had lost chromosomes during preparation of metaphases, although care was taken during analysis to exclude any obviously broken cells. Nevertheless, in preparations of LF strains prepared in the same way and scored by the same experienced cytogeneticist, a significant excess of hypoploidy occurred in five strains (126MA, 127MA, 128MA, 140MA, 142MA), and values for three other strains (107MA, 115MA, 121MA) were above the normal range. Only strains 126MA and 142MA showed excess hyperploidy, (47–79 chromosomes) and strains 127 MA and 142MA had excess 'polyploid' cells.

Four of the five strains whose abnormal cell frequencies were significantly above the normals exhibited levels of unstable structural aberrations that were also significantly above the normal level (126MA, 127MA, 140MA, 142MA). The frequency in 159MA was outside the normal range.

Four strains showed unusual features. Strain 126MA showed dramatically higher frequencies of structural aberrations at 99% PD compared to 76% PD and compared to any of the other strains. Although only 10 cells could be scored at 99% PD, all of these were grossly abnormal. These high levels of damage were comparable to those of late passage *TP53* heterozygous LF strains (Boyle et al. 1998). Strain 115MA was exceptional in that although most cells contained 45, 46 or 47 chromosomes, monosomy, trisomy and marker chromosomes were frequent with the result that virtually all cells had abnormal karyotypes, even at the earliest passage scored (50% PD). The same was true for early passage (39% PD) 140MA cells, which by 91% PD had evolved so that 49 of 64 (77%) of cells had 45 chromosomes and only 1 of 64 had 46 chromosomes. Strain 142MA also had a high frequency of cells with deletions and duplications of chromosomes.

These results consolidate the conclusion of our earlier study (Boyle et al, 1998) that chromosome instability, expressed mainly as increased hypoploidy, is a feature of LF cells from families without *TP53* mutations that may be a major factor contributing to the cancer proneness of these families. The present study emphasizes the point that genetic instability increases with cell age, and, in several cases, is increased dramatically just prior to senescence. However, there are also cases (79MA, 159MA) where this dramatic increase is not seen, implying that the expression of instability is heterogeneous within this class of LF strains. These observations are similar, but generally expressed less strongly, to those seen in LF cells carrying germline *TP53* mutations. The latter situation was associated with loss of the remaining wild-type p53 allele and in several cases was associated with clonal losses of chromosome segments that were observed by comparative genome hybridization (Burt et al, 2000). More detailed cytogenetic studies will be necessary to establish whether clonal events are also involved in the genetic instability of LF cells from families without *TP53* mutations, and whether the spectrum of clonal changes is the same in both LF groups.

In *TP53* heterozygous LF strains, genetic instability correlates well with resistance to the clonogenic killing effect of ionizing radiation (IR) given at low dose rate (LDR, ~1 cGy per min) (Sproston et al, 1996; Boyle, 1998). Radiation survival data for non-mutation strains is less complete, but strain 79MA (family 81) and two strains from family 80 (146MA, 154MA), comparable to 159MA, showed no resistance (Boyle et al 1998) and no genetic instability. In contrast, three strains (126MA, 127MA, 142MA) that are resistant to LDR IR (Boyle et al, 1998 and unpublished data) all show genetic instability. In LF cells with *TP53* mutations a similar correlation was found to be consistent with the hypothesis that such cells can tolerate a greater degree of genetic damage than normal cells, due to reduced genome surveillance (Lane, 1992; Williams et al, 1997).

In support of this hypothesis, genetic instability was also found to be associated with a compromised cell-cycle $G_1$ checkpoint in *TP53* heterozygous LF strains, but to be normal in cells from non-mutation families (Boyle et al, 1999). The possibility that other cell-cycle checkpoints may be compromised in non-mutation families is under examination and recently a germline mutation in the checkpoint kinase gene, *hChk2*, was identified in family 81 (Bell et al 1999) and is present in 79MA (JMV, unpublished results). So far we have been unable to detect an abnormal phenotype in fibroblasts associated with this mutation.

### ACKNOWLEFDGEMENT

This work was funded by the Cancer Research Campaign.

### REFERENCES

Bell D, Szydlo T, Kang D, Wahrer D, Shannon KE, Lubratovitch M, Versilis JJ, Isselbacher KJ, Birch JM, Varley JM, Li FP, Garber JE and Haber DA (1999) Heterozygous germline mutations in *hChk2l* are a cause of Li-Fraumeni syndrome. *Science* **286**: 2528–2531

Bischoff FZ, Yin SO, Pathak S, Grant G, Siciliano MJ, Giavanella BC, Strong LC and Tainsky MA (1990) Spontaneous abnormalities in normal fibroblasts from patients with Li-Fraumeni cancer syndrome: aneuploidy and immortalisation. *Cancer Res* **50**: 7979–7984

Boyle JM, Mitchell ELD, Greaves MJ, Roberts SA, Tricker K, Varley JM, Birch JM and Scott D (1998) Chromosome instability is a predominant trait of fibroblasts from Li-Fraumeni families. *Br J Cancer* **77**: 2181–2192

Boyle JM, Greaves MJ, Camplejohn RS, Birch JM, Roberts SA and Varley JM (1999) Radiation-induced $G_1$ arrest is not defective in fibroblasts from Li-Fraumeni families without *TP53* mutations. *Br J Cancer* **79**: 1657–1664

Burt EC, James LA, Greaves MJ, Birch JM, Boyle JM and Varley JM (2000) Genomic alterations associated with loss of heterozygosity for *TP53* in Li-Fraumeni Syndrome fibroblasts. *Br J Cancer* (in press)

ISCN (1995) *An international system for human cytogenetic nomenclature*. Mitelman F (ed) S Karger: Basel

Lane DP (1992) Cancer. P53, guardian of the genome. *Nature* **358**: 15–16

Sproston ARM, Boyle JM, Highway J, Birch JM and Scott D (1996) Li-Fraumeni fibroblasts are resistant to low dose-rate irradiation. *Int J Radiat Biol* **70**: 145–150

Varley JM, McGown G, Thomcroft M, Santibanez-Koref MF, Kelsey AM, Tricker KJ, Evans DGR and Birch JM (1997) Germline mutations of *TP53* in Li-Fraumeni families: an extended study of 39 families. *Cancer Res* **57**: 3245–3252

Williams KJ, Boyle JM, Birch JM, Norton JD and Scott D (1997) Cell cycle arrest defect in Li-Fraumeni syndrome: a mechanism of cancer predisposition? *Oncogene* **14**: 277–282

 *© 2000 Cancer Research Campaign*

